# Years of life lost and long-term outcomes due to glomerular disease in a Southeast Asian Cohort

**DOI:** 10.1038/s41598-023-46268-9

**Published:** 2023-11-05

**Authors:** Chitimaporn Janphram, Suchin Worawichawong, Montira Assanatham, Arkom Nongnuch, Sansanee Thotsiri, Umaporn Udomsubpayakul, Surangkana Wimolluck, Naparada Poomjun, Atiporn Ingsathit, Sinee Disthabanchong, Vasant Sumethkul, Wichai Aekplakorn, Panas Chalermsanyakorn, Chagriya Kitiyakara

**Affiliations:** 1 Somdech Phra Debaratana Medical Center, Faculty of Medicine Ramathibodi Hospital, Mahidol University, Bangkok, Thailand; 2 Department of Pathology, Faculty of Medicine Ramathibodi Hospital, Mahidol University, Bangkok, Thailand; 3 Department of Medicine Faculty of Medicine Ramathibodi Hospital, Mahidol University, Rama 6 Road, Bangkok, 10400 Thailand; 4 Department of Clinical Epidemiology and Biostatistics, Faculty of Medicine Ramathibodi Hospital, Mahidol University, Bangkok, Thailand; 5 Department of Community Medicine Faculty of Medicine Ramathibodi Hospital, Mahidol University, Bangkok, Thailand

**Keywords:** Nephrology, Kidney diseases, Glomerular diseases, Nephritis

## Abstract

Death and end-stage kidney disease (ESKD) are major outcomes of glomerular disease. (GD) The years of potential life lost (YLL) may provide additional insight into the disease burden beyond death rates. There is limited data on premature mortality in GD. In this retrospective observational cohort study, we evaluated the mortality, ESKD rates, and YLL in Thais with biopsy-proven GD. The mortality and combined outcome rates were determined by log-rank test and ESKD by using a competing risk model. YLL and premature life lost before age 60 were calculated for different GD based on the life expectancy of the Thai population. Patients with GD (n = 949) were followed for 5237 patient years. The death rate and ESKD rates (95%CI) were 4.2 (3.7–4.9) and 3.3 (2.9–3.9) per 100 patient-years, respectively. Paraprotein-related kidney disease had the highest death rate**,** and diabetic nephropathy had the highest ESKD rate. Despite not having the highest death rate, lupus nephritis (LN) had the highest YLL (41% of all GD) and premature loss of life before age 60. In conclusion, YLL provided a different disease burden assessment compared to mortality rates and identified LN as the major cause of premature death due to GD in a Southeast Asian cohort.

## Introduction

The global prevalence of CKD has increased markedly, resulting in considerable health and socioeconomic burden^1,2^. Glomerular disease (GD) is a major cause of CKD, contributing to a considerable portion of those requiring kidney replacement therapy for end-stage kidney disease (ESKD)^3^. GD patients have increased mortality due to complications of immunosuppressive treatment, kidney failure, or cardiovascular disease^4^. In ESKD registries, GDs are often referred to as a single clinical entity, but the term GD encompasses many diseases with considerable heterogeneity in progression rates to ESKD and mortality between different histological diagnoses^5^.

The distribution of GD types differs worldwide^5^. GD may contribute to even higher proportions of ESKD in low and middle-income countries, especially in patients under age 60^2,6^. Southeast Asia covers 8.5% of the world's population, with one of the largest burdens of CKD globally^7^. In recent times, Southeast Asia has seen a marked change in socioeconomic development that may impact the prevalence of infectious and non-communicable diseases affecting GD prevalence. Yet, there are limited current data exploring the medium to long-term outcomes of GD in Southeast Asia.

Despite death and ESKD being significant concerns of patients with GD^8^, the burden of specific GD remains to be thoroughly evaluated. Typically, the disease burden in GD has been measured by mortality rate, which does not consider the age of death. The number of deaths alone does not reflect the total burden on society, as some GD types harm younger people more than others. The number of years of potential life lost (YLL) estimates the average years a person would have lived if they had not died prematurely. YLL for a given disease depends on the age at death and the number of deaths at each age and may provide additional insight into the disease impact beyond data derived from death numbers alone^9,10^. YLL has been used to estimate disease burden in different types of cancers^9,10^, but there is limited YLL data in GD^11,12^ with no comparative data for different kinds of GD on premature mortality. Therefore, in this study, we evaluated the mortality, ESKD rates, and early life lost in different biopsy-proven primary and secondary GD in a Thai cohort.

## Results

**Figure 1:**
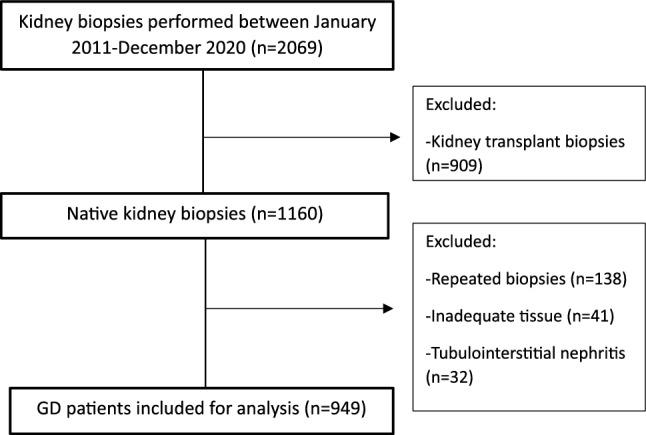
Study flow.

Of 2,069 biopsies performed, 909 were kidney allograft biopsies, 41 were inadequate, 32 were non-glomerular diseases, and 138 were repeat biopsies (Fig. 1). Nine hundred forty-nine patients with GD in their native kidneys were followed up for 76 (58, 97) months or 5237 patient-years. Baseline characteristics are shown in Table 1. The mean age at biopsy was 43 ± 17 years, and 59.4% were women. eGFR was 55 (25, 99) ml/min/1.73m^2^.

**Table 1:** Baseline characteristics by glomerular disease types. Data shown as Mean ± SD; Median (25,75 percentile).

	All GD (n = 949)	Primary GD (n = 473)	IgAN (n = 166)	FSGS (n = 130)	MN (n = 102)	MCD (n = 75)	LN (n = 254)	Pauci-immune (n = 21)	MPGN (n = 17)	IRGN (n = 13)	DN (n = 102)	Paraprotein-related kidney (n = 14)	CGN (n = 35)	Misc (n = 20)	*P* value
Age (years)	42 ± 17	43. ± 16	39 ± 15	46 ± 17	50 ± 14	43 ± 16	35 ± 14	63 ± 18	45 ± 20	46 ± 21	52 ± 14	58 ± 15	46 ± 16	39 ± 28	< 0.001
Males n (%)	382 (40.3)	229 (48.4)	74 (44.6)	60 (46.2)	52 (50.9)	43 (57.3)	33 (13.0)	9 (42.8)	11 (64.7)	8 (61.5)	59 (57.8)	7 (50)	21 (60)	5 (25)	< 0.001
SBP (mmHg) missing n (%)	131 ± 21 402 (42.3)	131 ± 18 199 (42)	131 ± 18 77 (46.3)	130 ± 18 50 (38.4)	132 ± 17 37 (36.2)	127 ± 18 35 (46.6)	132 ± 22 114 (44.8)	139 ± 20 5 (23.8)	153 ± 17 6 (35.2)	147 ± 27 5 (38.4)	141 ± 22 48 (47)	119 ± 21 5 (35.7)	144 ± 23 11 (31.4)	117 ± 17 9 (45)	0.001
Serum Creatinine (mg/dL) missing n(%)	1.4 (0.8, 2.5) 49 (5.1)	1.2 (0.8, 2.0) 27 (5.7)	1.6 (1.1, 2.5) 8 (4.8)	1.5 (0.9, 2.6) 6 (4.6)	0.9 (0.7, 1.2) 8 (7.8)	0.9 (0.7, 1.2) 5 (6.7)	1.0 (0.7, 2.0) 12 (4.7)	2.9 (1.4, 4.3) 1 (4.7)	1.7 (1.3, 2.5) 0	1.5 (1.0, 4.7) 0	2.7 (1.6, 4.6) 3 (2.9)	2.2 (1.1, 2.9) 1 (7.1)	3.4 (2.0, 6.6) 1 (2.8)	0.7 (0.6, 0.9) 4 (20)	< 0.001
eGFR (mL/min/1.73m^2^) missing n (%)	55 (25, 99) 49 (5.1)	62 (32, 96) 27 (5.7)	46 (24, 80) 8 (4.8)	47 (24, 87) 6 (4.6)	85 (59, 110) 8 (7.8)	87 (70, 107) 5 (6.7)	75 (35,114) 12 (4.7)	17 (10, 64) 1 (4.7)	44 (23, 57) 0	45 (12, 83) 0	23 (13, 42) 3 (2.9)	28 (17, 60) 1 (7.1)	15 (9, 33) 1 (2.8)	104 (81, 117) 4 (20)	< 0.001
Proteinuria (g/g Creatinine) missing n (%)	3.2 (1.4, 6.8) 142 (14.9)	2.8 (1.2, 6.2) 76 (16)	2.0 (1.0, 3.6) 25 (15)	3.0 (1.1, 7.2) 22 (21.4)	4.5 (2.1, 8.3) 14 (13.7)	3.5 (0.4, 7.0) 15 (20)	2.8 (1.4, 6.1) 30 (11.8)	2.3 (1.1, 6.2) 2 (9.5)	4.9 (1.8, 8.4) 1 (5.9)	2.8 (0.9, 5.4) 0	7.2 (4.2, 10.9) 16 (15.7)	5.1 (3.2, 18.8) 4 (28.5)	2.1 (1.0, 4.3) 5 (14.2)	1.3 (0.6, 3.9) 8 (40)	< 0.001

*CGN* Chronic glomerulonephritis; *DN* Diabetic nephropathy; *eGFR* estimated glomerular filtration rate; *FSGS* Focal segmental glomerulosclerosis; *GD* Glomerular disease; *IgAN* Ig A nephropathy; *IRGN* Infectious related glomerulonephritis; *LN* Lupus nephritis; *MCD* Minimal change disease; *MN* Membranous nephropathy; *MPGN* Membranoproliferative glomerulonephritis; *Misc* Miscellaneous (Alport, Thin basement membrane, IgM nephropathy); Primary GD = IgAN, FSGS, MN, MCD; *SBP* Systolic Blood Pressure.

Primary GD constituted 49.8% of all GD. Of the MN patients classified as primary GD, none had a history of malignancy on evaluation around the time of the biopsy. Of the FSGS patients classified as primary GD, none had HIV, severe obesity, or other identifiable secondary etiology of FSGS, although we did not perform genetic testing. Lupus nephritis (LN) was the most common (26.8%) GD type, followed by IgAN (17.5%) and FSGS (13.7%). There were statistically significant differences in age, sex, eGFR, and proteinuria between GD types. Age of onset was highest in pauci-immune GD and lowest in LN and IgAN. Serum Cr levels were lowest in MCD and MN and highest in CGN.

### Death

**Figure 2:**
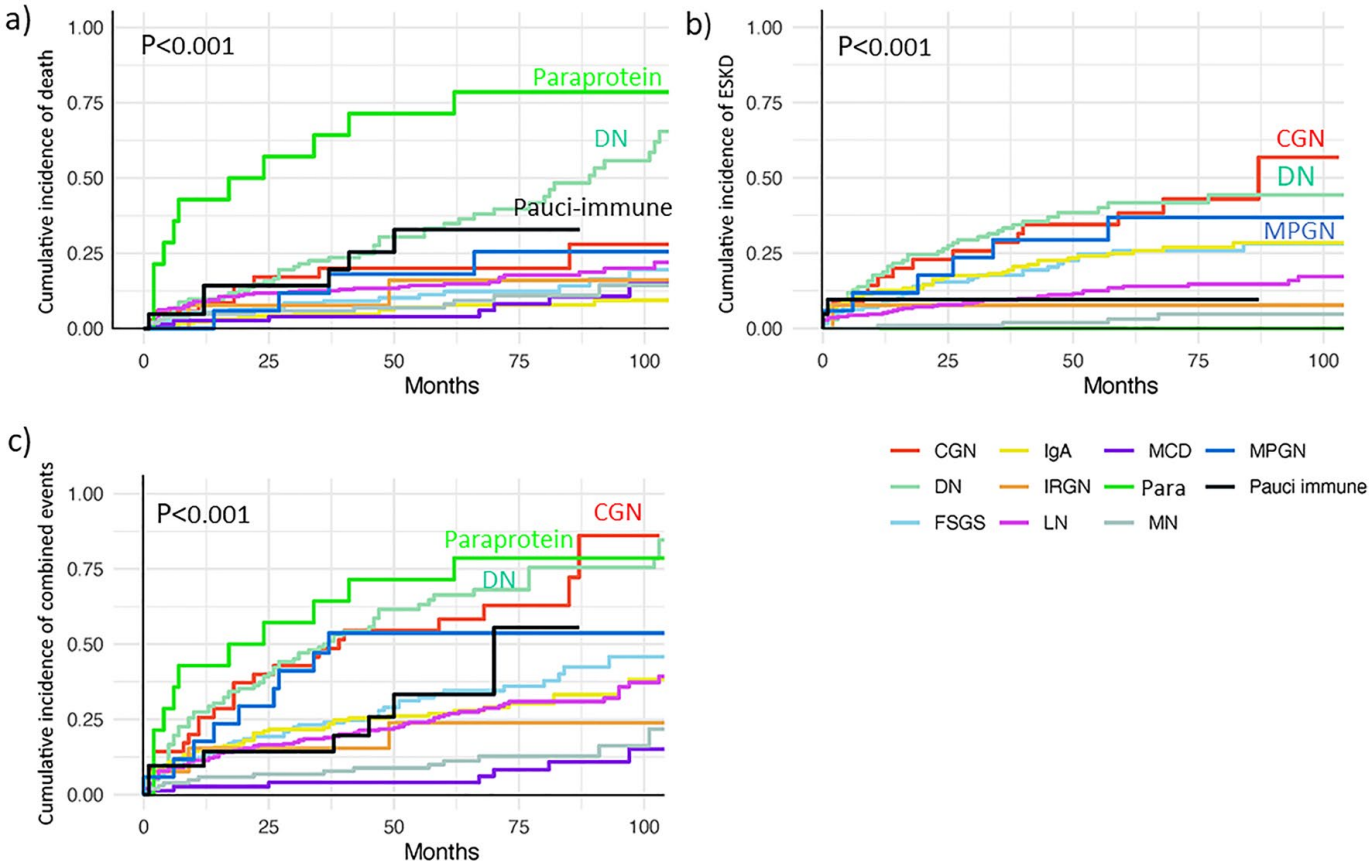
The cumulative incidence of outcomes for different GD (n = 949). (**a**) Death. (**b**) End-stage kidney disease (ESKD). (**c**) Combined events (death or ESKD).

Overall, 176 patients died (18.5%), and the death rate was 3.3 (95%CI 2.9–3.9) per 100 person-years (Table 2). Primary GD had lower deaths (10.8%) with an incidence rate of 1.8 (95%CI 1.4–2.4) per 100 person-years). Paraprotein-related kidney disease (MM) had the highest death rate, followed by diabetic nephropathy (DN). IgAN and MCD had the lowest rates. The incidence of death was significantly different between GD types (*P* < 0.001) (Fig. 2). Compared to MCD, the mortality risk was significantly increased for paraprotein-related kidney disease, DN, Pauci-immune glomerulonephritis and membranoproliferative glomerulonephritis (MPGN) (Table 3).

**Table 2:** Outcomes by glomerular disease types.

GD type (N)	Follow-up (Person years)	Follow-up (25,75 percentile) years	Deaths N (%)	Age at death (25,75 percentile) years	Mortality rate per 100 person-years (95%CI)	ESKD N (%)	ESKD rate per 100 person-years (95%CI)	Combined events N (%)	Combined events rate per 100 person-years (95%CI)
FSGS (130)	578.0	5.7 (3.4, 4.9)	19 (14.6)	64.0 (53.0, 75.0)	2.6 (1.6**–**4.0)	32 (24.6)	5.5 (3.9**–**7.8)	46 (35.4)	8.0 (6.0**–**10.6)
MCD (75)	475.0	6.5 (4.4, 8.2)	7 (9.3)	68.0 (40.0, 75.0)	1.5 (0.7**–**3.1)	0	0	7 (9.3)	1.5 (0.7**–**3.1)
IgA N (166)	791.2	6.1 (4.5, 7.9)	14 (8.4)	49.0 (34.3 ,67.5)	1.4 (0.8**–**2.3)	44 (26.5)	5.6 (4.1**–**7.5)	52 (31.3)	6.6 (5.0**–**8.6)
MN (102)	599.2	6.0 (4.9, 7.6)	11 (10.7)	64.0 (51.0 ,72.0)	1.8 (1.0**–**3.3)	5 (4.9)	0.8 (0.3**–**2.0)	15 (14.7)	2.5 (1.5**–**4.2)
LN (254)	1281.7	5.7 (4.3, 7.8)	46 (18.1)	36.5 (26.8, 53.3)	3.2 (2.4**–**4.3)	38 (14.9)	3.0 (2.2**–**4.1)	80 (31.5)	6.2 (5.0**–**7.8)
Pauci-immune (21)	75.1	3.7 (3.0, 4.9)	6 (28.6)	70.5 (65.0 ,76.8)	7.3 (3.3**–**16.3)	2 (9.5)	2.7 (0.7**–**10.6)	7 (33.3)	9.3 (4.4**–**19.6)
DN (102)	309.6	3.9 (3.0, 6.7)	46 (45.1)	59.0 (48.0 ,70.0)	9.6 (7.2**–**12.9)	42 (41.2)	14.0 (10.3**–**18.9)	70 (68.6)	22.6 (17.9**–**28.6)
MPGN (17)	73.3	6.2 (3.9, 8.2)	5 (29.4)	59.0 (48.0 ,71.5)	4.7 (2.0**–**11.3)	6 (35.3)	8.6 (3.9**–**19.2)	9 (52.9)	12.3 (6.4**–**23.6)
IRGN (13)	65.4	5.3 (4.3, 7.1)	2 (15.4)	47.5 (35.0 ,47.5)	2.8 (0.7**–**11.3)	1 (7.7)	1.5 (0.2**–**10.8)	3 (23.0)	4.6 (1.5**–**14.2)
CGN (35)	117.1	5.3 (3.2, 7.1)	8 (22.9)	44.0 (35.0 ,62.2)	4.4 (2.2**–**8.7)	14 (40)	12.8 (7.7**–**21.2)	23 (65.7)	19.6 (13.1**–**29.6)
Paraprotein-related kidney (14)	39.8	1.7 (0.3, 5.3)	11 (78.5)	67.0 (56.0, 71.0)	27.7 (15.3**–**50.0)	0	0	11 (78.5)	27.7 (15.3**–**50.0)
Primary GD (473)	2443.3	6.1 (4.4, 7.8)	51 (10.8)	64.0 (46.0 ,73.0)	1.8 (1.4**–**2.4)	81 (17.1)	3.3 (2.7, 4.1)	120 (25.4)	4.9 (4.1–5.9)
ALL GD (949)	4540.0	6.3 (4.8, 8.1)	176 (18.5)	56.0 (37.0 ,67.0)	3.3 (2.9**–**3.9)	186 (19.6)	4.2 (3.7, 4.9)	325 (34.2)	7.3 (6.6**–**8.2)

Data shown as Median (25,75 percentile).

*CGN* Chronic glomerulonephritis; *DN* Diabetic nephropathy; *FSGS* Focal segmental glomerulosclerosis; *GD* Glomerular disease; *IgAN* Ig A nephropathy; *IRGN* Infectious related glomerulonephritis; *LN* Lupus nephritis; *MCD* Minimal change disease; *MN* Membranous nephropathy; *MPGN* Membranoproliferative glomerulonephritis; *Misc* Miscellaneous (Alport, Thin basement membrane, IgM nephropathy); Primary GD= IgAN, FSGS, MN, MCD.

**Table 3:** Risk of outcomes by glomerular disease types.

GD type (N)	Death HR (95% CI)	Combined death and ESKD HR (95% CI)	ESKD Subdistribution HR (95% CI)
MCD (75)	Ref	Ref	Undetermined
FSGS (130)	1.7 (0.7–4.1)	**5.3** **(2.2–12.5)**	**5.7** **(2.3–14.5)**
IgAN (166)	0.9 (0.4–2.3)	**4.4** **(1.9–10.3)**	**5.2** **(2.1–13.1)**
MN (102)	1.2 (0.4**–**3.2)	1.7 (0.6**–**4.3)	Ref
LN (254)	2.1 (0.9**–**4.9)	**4.5** **(2.0–12.2)**	**2.7** **(1.1–6.7)**
Pauci-immune (21)	**3.9** **(1.1–13.2)**	**4.9** **(1.4–16.1)**	1.2 (0.1–10.7)
DN (102)	**7.9** **(3.3–19.1)**	**21.3** **(8.8–51.4)**	**10.8** **(4.3–27.1)**
MPGN (17)	**4.0** **(1.1–14.8)**	**10.9** **(3.2–37.4)**	**7.4** **(2.2–25.0)**
IRGN (13)	1.8 **(0.3–9.6)**	2.9 (0.6**–**13.1)	1.7 (0.2**–**15.4)
CGN (35)	2.9 **(0.9–8.7)**	**18.6** **(6.5–52.9)**	**10.1** **(3.7–27.5)**
Paraprotein-relatedkidney (14)	**35.6** **(7.9–158.8)**	**35.6** **(8.0–158.8)**	Undetermined

*CGN* Chronic glomerulonephritis; *DN* Diabetic nephropathy; *FSGS* Focal segmental glomerulosclerosis; *GD* Glomerular disease; *IgAN* Ig A nephropathy; *IRGN* Infectious related glomerulonephritis; *LN* Lupus nephritis; *MCD* Minimal change disease; *MN* Membranous nephropathy; *MPGN* Membranoproliferative glomerulonephritis; *Misc* Miscellaneous (Alport, Thin basement membrane, IgM nephropathy); Primary GD = IgAN, FSGS, MN, MCD.

Bold denotes statistical significance.

### ESKD

Overall, 186 patients (19.6%) had ESKD, and the ESKD rate was 4.2 (95%CI 3.7–4.9) per 100 person-years (Table 2). DN had the highest rate of ESKD, followed by chronic glomerular disease (C). The ESKD rate in primary GD was 3.3 (95%CI 2.7–4.1) per 100 person-years, with IgAN having the highest ESKD rate among primary GD. No ESKD occurred in paraprotein-related kidney disease or MCD. GD types showed different cumulative ESKD rates (*P* < 0.01) (Fig. 2). Using death as a competing risk, DN, CGN, MPGN, FSGS, IgAN, and LN had increased risk of ESKD with MN as the reference (Table 3).

### Combined events

Overall, 34.2% developed death or ESKD. The combined event rate for all GD was 7.3 (95%CI 6.6–8.2) per 100 person-years (Table 2), with the highest rate being paraprotein-related kidney disease, followed by DN and CGN. The combined event rate was 4.9 (95%CI 4.1–5.9) per 100 patient-years in primary GD, with FSGS having the highest rate among primary GD. GD types significantly differed in cumulative combined events (*P* < 0.001) (Fig. 2). Compared to MCD, the risk for combined events was significantly increased for all GD types except for MN and infections related-glomerulonephritis (IRGN) (Table 3).

### Years of life lost

#### Median YLL

**Figure 3:**
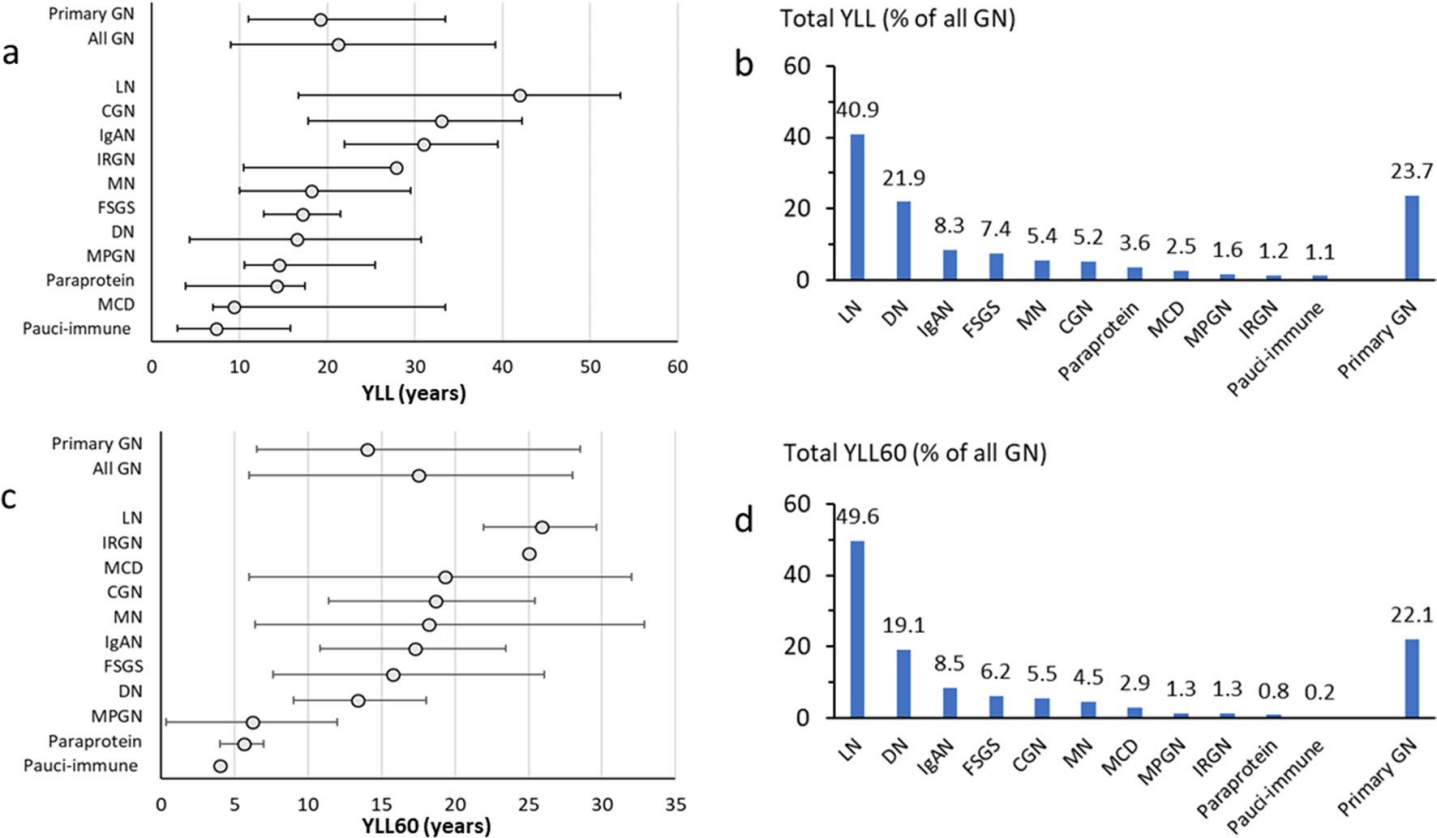
Years of life lost (YLL) by GD types (including diabetes). (**a**) All patients: Median YLL. Open circle denotes median with ends representing 25 and 75 percentile. (**b**) All patients: Proportion of total YLL. (**c**) Premature death before age 60: Median YLL60. Open circle denotes median with ends representing 25 and 75 percentile. (**d**) Premature death before age 60: Proportion of total YLL60.

The age at death for GD is shown in Table 2. The median YLL for those who died were: 21.2 (12.2, 39.2) and 19.2 (8.2, 33.5) years in all GD and primary GD, respectively. The median YLL of GD types is shown in Fig. 3a. LN had the highest median YLL (42.0 (26.2, 52.5) years, followed by CGN (33.0 (15.2, 42.2)) years and IRGN.

#### Total YLL of each GD

The total YLL was 4,492.7 years for all GD and 1,066 for primary GD (23.7% of all YLL) (Table 4). The total YLL for each GD type is shown as a percentage of the total YLL for all GD (Fig. 3b). LN accounted for over 40% of the total YLL of all GD, followed by DN (22%) and IgAN (8.3%).

**Table 4:** Years of life lost for all ages and premature life lost before age 60 by GD type.

GD type	All patients		Disease onset before Age 60	
	N	Total YLL (years)	N	Total YLL60 (years)
Pauci-immune	21	52.1	7	4
FSGS	130	331.8	98	126
IgAN	166	371.8	148	173
LN	254	1841.5	237	1009
MN	102	246.3	75	91
MPGN	17	73.4	13	25
MCD	75	116.1	63	58
IRGN	13	55.9	8	25
CGN	35	234.6	26	112
Paraprotein-related kidney	14	160.9	5	17
Miscellaneous	20	25.5	17	5
All GD (excluding DN)	847	3509.9	69	1645
DN	102	983.4	70	388
All GD (including DN)	949	4493.3	767	2033
Primary GD	473	1066.0	384	448

*YLL* years of life lost; *YLL60* years of life lost before age 60; *CGN* Chronic glomerulonephritis; *DN* Diabetic nephropathy; *FSGS* Focal segmental glomerulosclerosis; *GD* Glomerular disease; *IgAN* Ig A nephropathy; *IRGN* Infectious related glomerulonephritis; *LN* Lupus nephritis; *MCD* Minimal change disease; *MN* Membranous nephropathy; *MPGN* Membranoproliferative glomerulonephritis; *Misc* Miscellaneous (Alport, Thin basement membrane, IgM nephropathy); Primary *GD* IgAN, FSGS, MN, MCD.

### Premature death before age 60

Of 767 patients biopsied before age 60, 110 died before age 60. The total YLL before age 60 (YLL60) for all GD was 2033 years (including DN) with a median YLL of 17.4 years (Table 4). For primary GD, the total YLL60 was 448 years, with a median YLL60 of 14 years. LN had a median YLL60 of 26 years and the largest burden of premature death with a total YLL60 of 1009 years (49.6% of total YLL60 all GD) (Fig. 3c,d).

## Discussion

We evaluated the medium-term outcomes of biopsy-proven GD in a real-world setting in a cohort of Thai subjects with 5237 patient-years follow-up. The novel findings were considerable differences in mortality, ESKD rates, and years of life lost among different GD. There were discrepancies in the relative impact of different GD when YLL was evaluated instead of mortality rates. Paraprotein-related kidney disease, DN and pauci-immune glomerulonephritis had the highest mortality, whereas CGN and DN had the highest ESKD rates. MCD had the lowest combined and ESKD rates overall. Despite not having the highest death rate, LN had the highest YLL, contributing to almost half of the premature loss of life before age 60, with IgAN and FSGS also important.

The frequency of different GD varies worldwide due to differences in genetic, environmental factors, and biopsy selection practices^6^. In our series, LN was the commonest GD, followed by IgAN, FSGS, MN, and MCD. A recent report from China showed a similar prevalence of MN, IgAN, and MCD, but LN and FSGS were much less common^13^. Our contemporary data showed differences from an earlier study from Thailand^14^. Between 1982 and 2005, LN and IgM nephropathy were the most common GD (27% each), followed by IgAN (10.8%), whereas only a few cases were diagnosed with MCD. By contrast, we only reported 2 cases of IgM nephropathy and a sizable proportion of MCD. More stringent criteria for diagnosis of IgM nephropathy, including the requirement for electron-dense deposits, may account for higher numbers of MCDs in our study^15^. A similar time trend of reduced diagnosis of mesangial proliferative glomerulonephritis accompanied by an increase in MCD was also found in Singapore^16^.This reduced mesangial proliferation might be due to decreased systematic antigen challenge from infectious agents due to economic development. Consistent with this notion, the prevalence of IRGN in our study was three folds lower than in the previous era^13^. Among other observed trends, FSGS was the third most common GD (13%) in our series, whereas the overall FSGS rate was only 1.7% in the previous decades^13^. This finding is consistent with the global increase in FSGS^16,17^.

Paraprotein-related kidney diseases encompass cast nephropathy, often associated with variable glomerular involvement and other manifestations of light chain deposition^18^. In our study, secondary GD, such as paraprotein-related kidney disease, and pauci-immune glomerulonephritis (including ANCA-associated) had the highest mortality rates reflecting the older age of onset, disease severity, and the complications of cytotoxic regimen in these diseases^18,19^. The lower ESKD rate likely reflects that for ESKD to be registered, the patient must be on dialysis for over three months. Patients who died or stopped dialysis before the three-month duration would not have been documented as ESKD. In our cohort, MPGN was typically associated with HCV infections; hence, the condition was not classified as a primary GD. Poor prognosis for MPGN is consistent with previous studies^20^.

The increase in DN in our series compared to the previous era may be due to increasing diabetes prevalence and more standardized indications for biopsies similar to other global regions^6,15^. Since kidney biopsies were performed only in patients with unusual features such as massive proteinuria or rapid disease progression, high kidney failure and mortality rates in DN may reflect the advanced stage of kidney disease and concomitant cardiovascular risk factors already present at the time of biopsy^21^.

Several studies from lower to middle-income countries have examined the prevalence of various types of GD in cross-sectional research or individual GD types over time^6^. However, there are few recent studies covering the period since the year AD 2000 that use biopsy registry data to compare outcomes in patients with both primary and secondary GD. The available data often underscores the inferior outcomes associated with secondary GD. For instance, in mostly Caucasian patients from New Zealand, rapidly progressive glomerulonephritis was the leading cause of ESKD and mortality, MCD showed the lowest risk, whereas FSGS and IgAN posed intermediate risks^22^. The proportion of patients who developed outcomes was comparable to our study. Further, similar trends—particularly the heightened risk of rapidly progressive glomerulonephritis relative to other primary GD types regarding ESKD and mortality outcomes—have been observed in biopsy series from Denmark and Austria^23,24^.Notably, these Caucasian cohorts had a significantly lower proportion of lupus nephritis than our study group. Another study on Singaporeans over 60 showed that the risk of combined outcomes was over tenfold higher for LN or DN than IgAN^25^.

Among primary GD, we found that FSGS had the highest combined event rate with almost double the mortality rate of IgAN but comparable ESKD rates. MN had intermediate risk, whereas MCD had negligible ESKD risk, but some patients still died. Other recent biopsy series focusing on primary GD from Taiwan^26^ and Korea^20^ and a study using electronic health records from the USA^27^ found the highest ESKD risk with FSGS and the lowest rates for MCD, similar to our study, but secondary GD was not evaluated in these studies.

In 2017, CKD resulted in an estimated 28·5 million YLL globally, with Southeast Asia having one of the highest burdens of CKD^28^. However, comparative data on YLL in different GD has been limited. Assessment of YLL highlighted LN as a major burden in Thai patients, although LN did not have the highest mortality rate per case. LN was the most common GD type. The younger disease onset means that deaths related to disease activity and immunosuppression had greater effects on YLL than other GD with older age of onset. Although no previous studies in biopsy-proven LN, one study evaluated patients with SLE in Oslo^11^. The premature years of life lost before age 60 for SLE patients with a history of kidney involvement were 21 per 1000 person-years. As a major cause of global CKD, DN also showed a substantial burden as a proportion of YLL. IgAN and FSGS considerably impacted YLL, with a greater contribution of IgAN in premature mortality before age 60. A recent study reported the YLL of IgAN patients from the Southeastern United States to be about 10.1 years^12^. A greater reduction in life expectancy of 26.5 years in our IgAN patients may result from the shorter follow-up duration, leading to an overestimation of premature YLL since it did not capture later premature deaths or potential differences in disease severity at presentation or subsequent therapy.

To our knowledge, this is the first study to evaluate the prematurity of death as measured by years of life lost in diverse GD types and the first contemporary study of intermediate to long-term outcomes in a Southeast Asian population. The strength of this study is that all diagnoses were based on kidney biopsy and included both primary and secondary GD allowing direct comparisons between GD types. Hard outcomes were obtained from official national databases enabling accurate ascertainment of outcome dates. Therefore, events occurring in patients who defaulted on follow-up visits at our hospital were still captured. The outcome rates of our study reflect real-world data comprising of those who were followed up at our Institution, and those followed up elsewhere. The risk of ESKD was calculated using competing risk analysis. This study has several implications, especially highlighting the importance of YLL in addition to mortality rates as an assessment of the disease burden in GD around the world. The finding that LN, IgAN, and FSGS are major causes of premature death in Thai patients will have implications for resource allocation and the design of prevention programs to target these diseases early on. This study also confirmed a high burden of diabetic nephropathy. Nonetheless, because most patients with diabetic kidney diseases are not biopsied, the entire burden of diabetic kidney disease cannot be assessed.

There are several limitations. First, although our hospital is one of the largest referral centers receiving patients from all over Thailand, this is a single-center study, which may be subjected to bias in outcome rate estimation, especially for rarer diseases. Second. although we performed standard investigations to exclude systemic causes, it is possible that in some cases of primary GD, such as MN or FSGS, an underlying disease, such as occult malignancy with late presentation or genetic abnormalities, could be an underlying etiological factor. Nonetheless, the proportion of primary GD cases misclassified as secondary GD is likely small. Third, we did not verify whether patients had undergone kidney biopsies before 2011. Repeat biopsies are not typically done during routine follow-ups in our center, although some patients with LN or MCD might have had an earlier biopsy, and the biopsy included in this study could have been taken during a relapse or due to treatment resistance. While this could lead to an underestimation of disease onset in a few patients, the overall number of such cases is small and is unlikely to affect the estimation of disease incidence or outcomes in a major way. Fourth, some patients presented with the diagnosis of CGN, which may represent an advanced stage of other GD. This may lead to an underestimation of the disease impact of some GD types. Fifth, a kidney biopsy may not have been performed in patients with advanced disease or mild diseases (e.g. isolated microscopic hematuria). Therefore, although our study provides a valuable estimate of the relative impact of different GD types, the actual burden of GD types may not be fully ascertained. This limitation is common to other biopsy series. Furthermore, in our study, we calculated the YLL due to premature mortality occurring within a median follow-up period of 76 months after the diagnosis of glomerular disease. We did not consider YLL resulting from premature deaths that might happen in the later stages after diagnosis. It is essential to recognize that individuals with glomerular disease may have a shorter life expectancy compared to the general population^12,29,30^. As a result, it has the potential to result in an overestimation of the median YLL within our study cohort, particularly in cases of glomerular diseases diagnosed at a younger age. However, our evaluation of the median YLL was carried out within the context of examining the YLL distribution across different types of glomerular disease. Finally, we do not have data on the cause of death. Early deaths may be related to systemic disease activity or treatment complications, whereas later deaths may be due to cardiovascular disease^31^. Dialysis in Thailand is covered under universal coverage. Therefore, deaths due to the inability to access kidney replacement therapy are unlikely to be a major contributor.

In conclusion, GD results in a considerable healthcare burden on the Thai population. The relative frequency and impact of GD types are similar but not identical to other East Asian countries. Evaluation of YLL identified LN, and to a lesser extent, DN, FSGS, and IgAN as major causes of early death due to GD. Future research utilizing larger multicenter cohorts and extended follow-up durations could provide a more comprehensive understanding of the burden GD poses to both the Thai and global communities.

## Methods

### Study design

This is a retrospective observational cohort study from a tertiary referral center in Bangkok, Thailand. We obtained data from the Ramathibodi Hospital Glomerular Registry database of all patients who had a kidney biopsy between 1st January 2011 to 31st December 2020. All participants were enrolled on the biopsy date and followed up until the end of the study or death. This study conformed to the Declaration of Helsinki and was approved by the Faculty of Medicine, Ramathibodi Hospital Ethics Committee (ID MURA2022/200).

### Study population

Kidney biopsies were performed for clinical indications. All patients gave informed written consent. Patients aged > 15 years with GD in their native kidneys were included. We excluded patients with kidney allografts, without GD, or those with inadequate tissue cores. Only data from the first diagnostic biopsy was used in patients with repeated biopsies.

Baseline characteristics were recorded on the day or within one month of the biopsy. Routine laboratory investigations, including urinalysis and specific serologies, were performed. Proteinuria was evaluated from the urine protein creatinine ratio. The estimated glomerular filtration rate (eGFR) was calculated from enzymatic serum creatinine by the Chronic Kidney Disease Epidemiology Collaboration equation (CKD-EPI) for non-blacks^32^ according to the recommendations of the Nephrology Society of Thailand.

### Histology

A specialist nephropathologist assigned each type of GD diagnosis based on light microscopy, immunofluorescence, and electron microscopy (EM) according to standard criteria^33^. Kidney biopsy cores were fixed in Glyo-Fixx (Thermo Scientific, USA), embedded in paraffin, and cut into Sects. (2 µm), and tissues stained by hematoxylin–eosin, Masson trichrome, periodic acid-Schiff, and Jones methenamine silver stains.

We categorized patients into 12 GD types, including Chronic glomerulonephritis (CGN) and Miscellaneous. Chronic glomerulonephritis (CGN) was defined as advanced global sclerosis of all glomeruli^34^ without positive immunofluorescence staining, and a specific diagnosis could not be made. The miscellaneous group comprised a heterogeneous group of diseases, including IgM nephropathy, Alport’s syndrome, and Thin basement membrane disease.

GD types were classified into primary and secondary GD based on the presence or absence of systemic conditions based on medical record reviews, laboratory investigations, and nephrologist diagnoses. Primary GD comprised a combined group of IgA nephropathy (IgAN), membranous nephropathy (MN), focal segmental glomerulosclerosis (FSGS), and minimal change disease (MCD)^35,36^. We categorized all patients with clinical, laboratory, or histological features consistent with lupus membranous nephritis as lupus nephritis (LN).

### Follow-up and outcomes

Patients were treated according to physician preferences based on the 2012 KDIGO guideline^37^. Outcomes were obtained until death or 31st December 2021. The primary outcome was *death*. The secondary outcomes were: *end-stage kidney disease* (ESKD) or *combined death or ESKD*. The date of death was obtained from the National Death Registry, Ministry of Health. ESKD was defined as the need for long-term kidney replacement therapy. The start date was obtained from the Nephrology Society of Thailand ESKD registry, which includes all patients on chronic dialysis (> 90 days) or who had a kidney transplant in Thailand.

### Years of life lost

The years of life lost (YLL) were calculated for each person who died (expected age − actual age at death)^10,38^. The expected age of death was estimated from the life expectancy of the Thai population for the individual’s age and sex using data from the Institute for Population and Social Research^39^ such that if patients died before the age of 60, men had an expectancy age of 77.4 years and women had an expectancy age of 83.2 years; if patients died between the ages of 60 and 80 or older, men had an expected age of 86.1 years and women had an expectancy age of 88.4 years.

The *total YLL* of each GD type was the summation of the years lost by patients in each type^9^. The *median YLL* was the 50th percentile of the YLL for all people who died in each GD type.

### Premature life lost before age 60

We calculated the years of early life lost before age 60 (YLL60) in a subgroup biopsied before age 60, using age 60 as the life expectancy age.

### Statistical analysis

Baseline characteristics and outcomes were assessed for each type of GD, all GD, and primary GD. Missing data were excluded from analysis. Normally distributed variables were expressed by mean (± SD), and groups were compared by one-way ANOVA with Bonferroni correction. Non-normally distributed variables were described by median (25,75 percentile) and groups were compared by the Kruskal–Wallis test, followed by Dunn's test for comparisons of specific group pairs.

Categorical data were expressed as frequencies (%) and compared by Chi-square tests. Survival analysis was performed to calculate incidence rates per 100 person-years of ESKD and mortality rate for each GD^27^. For death or combined outcome of death or ESKD, we constructed exposure–outcome models for survival using Kaplan–Meier curves, log rank test, and Cox proportional hazard regression for comparison among types of GD with MCD as the reference. For ESKD, we used competing risk analysis and calculated the subdistribution hazard model (Fine-Gray), treating death as a competing event with MN as the reference as no ESKD occurred in MCD^40^.

To account for the competing risk, subdistribution hazard ratios were calculated using the command “stcrreg” of Stata program version 16 (Statacorp, College Station,Tx,USA). Statistically significance was defined by a two-sided *P*-value at 0.05.

## Data Availability

Data available upon request by contacting Chagriya Kitiyakara. (Email: Kitiyakc@yahoo.com).
